# The I-TevI Nuclease and Linker Domains Contribute to the Specificity of Monomeric TALENs

**DOI:** 10.1534/g3.114.011445

**Published:** 2014-04-16

**Authors:** Benjamin P. Kleinstiver, Li Wang, Jason M. Wolfs, Tomasz Kolaczyk, Brendon McDowell, Xu Wang, Caroline Schild-Poulter, Adam J. Bogdanove, David R. Edgell

**Affiliations:** * Department of Biochemistry, Schulich School of Medicine and Dentistry, Western University, London, Ontario, Canada N6A 5C1; † Department of Plant Pathology and Plant-Microbe Biology, Cornell University, 334 Plant Science, Ithaca, New York 14853; ‡ Robarts Research Institute, Schulich School of Medicine and Dentistry, Western University, London, Ontario, Canada N6A 5B7

**Keywords:** monomeric TALEN, GIY-YIG nuclease, I-TevI, TAL effector, genome editing

## Abstract

Precise genome editing in complex genomes is enabled by engineered nucleases that can be programmed to cleave in a site-specific manner. Here, we fused the small, sequence-tolerant monomeric nuclease domain from the homing endonuclease I-TevI to transcription-like activator effectors (TALEs) to create monomeric Tev-TALE nucleases (Tev-mTALENs). Using the PthXo1 TALE scaffold to optimize the Tev-mTALEN architecture, we found that choice of the N-terminal fusion point on the TALE greatly influenced activity in yeast-based assays, and that the length of the linker used affected the optimal spacing of the TALE binding site from the I-TevI cleavage site, specified by the motif 5′-CNNNG-3′. By assaying activity on all 64 possible sequence variants of this motif, we discovered that in the Tev-mTALEN context, I-TevI prefers A/T-rich triplets over G/C-rich ones at the cleavage site. Profiling of nucleotide requirements in the DNA spacer that separates the CNNNG motif from the TALE binding site revealed substantial, but not complete, tolerance to sequence variation. Tev-mTALENs showed robust mutagenic activity on an episomal target in HEK 293T cells consistent with specific cleavage followed by nonhomologous end-joining repair. Our data substantiate the applicability of Tev-mTALENs as genome-editing tools but highlight DNA spacer and cleavage site nucleotide preferences that, while enhancing specificity, do confer moderate targeting constraints.

Genome editing in complex genomes is enhanced by the introduction of a double-strand break (DSB) or single-strand nick at the targeted locus by site-directed nucleases (Bibikova *et al.* 2003; Porteus and Baltimore 2003). Recent years have witnessed an explosion in the development of genome-editing tools (Bogdanove and Voytas 2011; Kleinstiver *et al.* 2012; Mali *et al.* 2013b; Pennisi 2013; Schierling *et al.* 2012; Shalem *et al.* 2014; Takeuchi *et al.* 2011), each with its own benefits and constraints (Halford *et al.* 2011). Zinc-finger nucleases (ZFNs) and TAL effector nucleases (TALENs) are two widely used reagents (Christian *et al.* 2010; Kim *et al.* 1997; Li *et al.* 2011). The DNA-binding domain of TAL effectors (TALEs) consists of up to 30 tandem repeats of a 33-amino acid to 35-amino acid motif with two variable residues [termed the repeat variable di-residues (RVDs)] that confer single nucleotide specificity to each repeat so that the repeats linearly define the nucleotide sequence of the binding site (Boch *et al.* 2009; Moscou and Bogdanove 2009). The one-to-one correspondence of RVD to DNA base makes TALENs more readily programmed for new specificities than ZFNs are, and has led to their widespread adoption (Hockemeyer *et al.* 2011; Reyon *et al.* 2011). TALENs and ZFNs incorporate the nuclease domain of the type IIS restriction enzyme FokI, and the requirement of the FokI nuclease domain to dimerize for activity necessitates the design, construction, and delivery of TALENs (or ZFNs) in pairs targeting opposing sites in a head-to-head orientation flanking the target for cleavage. (Bitinaite *et al.* 1998; Smith *et al.* 2000). The CRISPR/Cas9 system is a more recent, and now also a widely adopted tool (Burgess 2013). In this system, the Cas9 nuclease is guided to its target via interaction with an RNA molecule bearing a sequence complementary to the target. Targeting specificity is governed by the hybridization of the RNA with the DNA target and is limited to 17 to 20 bps (Fu *et al.* 2014). To improve specificity, CRISPR/Cas9 nickases have been developed that must operate in pairs in much the same way as TALENs (Cong *et al.* 2013; Mali *et al.* 2013a,b), yet these are not as efficient and add moderately to the complexity of implementation.

To eliminate the complexity of the FokI and nickase-based platforms while retaining the potential for high specificity, we recently developed novel genome-editing reagents that function as monomers by fusing the small, sequence-tolerant nuclease domain of the GIY-YIG homing endonucleases (GIY-HEs) to customizable DNA-binding proteins (Kleinstiver *et al.* 2012). By fusing the nuclease domain and linker of the GIY-HE I-TevI to zinc fingers (ZFs) and LAGLIDADG homing endonucleases (LHEs), we created monomeric Tev-ZFEs and Tev-LHEs that cleave sequences defined by the ZF or LHE binding domains. These nucleases require a 5′-CNNNG-3′ motif spaced at an appropriate distance from the binding site for efficient cleavage, because mutation of the CNNNG motif abolished cleavage by the Tev-LHE or Tev-ZFEs. This behavior mimics that of the wild-type I-TevI enzyme on its native DNA substrate derived from the thymidylate synthase (*td*) gene of phage T4 (Bryk *et al.* 1995). The additional specificity of the I-TevI nuclease domain has the potential to reduce cleavage at off-target sites, because the required cleavage motif may not be found within the vicinity of sites that result from promiscuous DNA binding. However, utility of Tev-ZFEs is constrained by the imperfect programmability of the ZF proteins, and extensive engineering is required to alter LHE specificity.

To create monomeric nucleases with greater DNA-targeting potential, we constructed monomeric Tev-TALENs (Tev-mTALENs) by fusing different lengths of the I-TevI nuclease domain to the N-terminus of TALEs. Our constructs are similar to recently described compact TALENs (cTALENs) (Beurdeley *et al.* 2013), but here we report key differences in DNA spacer and cleavage motif requirements and overall activity relative to those reported in the cTALEN study. In particular, using an *in vitro* assay with purified protein, we demonstrate that the requirement of an appropriately spaced 5′-CNNNG-3′ cleavage motif is preserved within the Tev-mTALEN architecture. Furthermore, by interrogating *in vivo* cleavage activity on all 64 possible NNN triplets at this site, and on a library of substrates with random DNA spacer sequences, we define clear nucleotide preferences for optimal activity and a moderate influence of spacer sequence. These cleavage site and DNA spacer sequence dependencies enhance targeting specificity yet confer moderate targeting constraints. Finally, we demonstrate that Tev-mTALENs function on an episomal target in HEK293T cells. Together, our data validate Tev-mTALENs as a useful addition to the toolbox for efficient genome editing but reveal the need for judicious target selection and further elucidation of the influence of the spacer sequence.

## Materials and Methods

### Bacterial and yeast strains

*Escherichia coli* DH5α and ER2566 (New England Biolabs) were used for plasmid manipulations and protein expression, respectively. *E. coli* strains were grown in Luria-Broth media supplemented with the appropriate antibiotics. *Saccharomyces cerevisiae* strains YPH500(α) and YPH499(a) were used for the single-strand annealing assay and grown in appropriate media as described (Christian *et al.* 2010).

### Construction of Tev-mTALENs and substrate plasmids

Substrates for Tev-mTALENs were constructed by first cloning oligonucleotides corresponding to the target site into the XbaI/SphI sites of pTox. Each substrate, differing in the DNA spacer length, was PCR-amplified with flanking primers and cloned into the BglII/SpeI sites of the yeast vector pCP5.1 (Christian *et al.* 2010) to create the TP series of plasmids (TP5-TP34) for the yeast activity assay. Substrates for use in HEK 293T cells were constructed in the same manner and cloned into the SacI/XhoI sites of pcDNA3(+) (Life Technologies). Tev-mTALENs were first constructed in pACYC (New England Biolabs) by changing the NcoI site to PciI and by inserting a stop codon downstream of the BglII site, and the full-length PthXo1 TAL effector (Yang *et al.* 2006) was then cloned into the BamHI/BglII sites. The I-TevI nuclease domain and various linker lengths were then cloned into the PciI/BamHI sites. Tev-mTALENs that differed in the N-terminal fusion point were constructed by first removing the N-terminal BamHI/SphI fragment from PthXo1, leaving the RVD-containing repeats intact. PCR products corresponding to the new N-terminal fusion point were then cloned into the BamHI/SphI sites, and the I-TevI nuclease domain was cloned into the PciI/BamHI sites. For yeast assays, each Tev-mTALEN construct was digested with PciI/XhoI and subcloned into the XhoI/SalI sites of pGPD423 (Alberti *et al.* 2007). For mammalian assays, the pACYC backbone was first modified by including an RsrII site upstream of the PciI site, and then Tev-mTALEN constructs were inserted as above. Tev-mTALENs were subsequently cloned into the PstI/RsrII sites of pExodus. A list of Tev-mTALENs tested for activity is found in Supporting Information, Table S1 and Figure S1.

### Cleavage mapping

Mapping of Tev-mTALEN sites used N169-T120 or S206-D1 Tev-mTALENs that were purified untagged. Briefly, untagged Tev-mTALEN constructs in pACYC-Duet were overexpressed in *E. coli* ER2566, and the cells were suspended in buffer A (20 mM Tris-HCl pH 7.5, 200 mM NaCl, 1 mM DTT, 5% glycerol, and 0.1 mM EDTA) and lysed with a cell homogenizer (Avestin). Clarified extracts were applied to a Hi-Trap Heparin column (GE Healthcare) equilibrated in the same buffer and eluted with a linear gradient of 200 mM to 1 M NaCl. Fractions containing 250 to 325 mM NaCl were pooled, dialyzed, and applied to a SP-FF column (GE Healthcare) equilibrated in buffer A and eluted in steps of 200 nM NaCl to a final concentration of 1 M NaCl. The 400-mM elutions were pooled and applied to a FF-Q column (GE Healthcare) equilibrated in buffer A and eluted in steps of 200 mM NaCl. The 400-mM fractions were pooled, concentrated to 0.5 ml, and loaded onto a 30-ml Superose 12 gel filtration column (GE Healthcare) equilibrated in buffer A and 0.25-ml fractions collected over 1 column volume (Figure S4). Endonuclease assays on substrates with different length spacers used oligonucleotides end-labeled at the 5′ end with T4 polynucleotide kinase and ^32^P-γATP prior to annealing. Cleavage reactions were incubated for 10 min at 37° in 20-μl reaction volumes in 1× NEBuffer 3 and were resolved on 10% denaturing urea-polyarcylamide gels. Mapping of cleavage sites used supercoiled pSP72-TP15 in 20 μl of 1× NEBuffer and a five-fold molar excess of protein to DNA. Linear cleavage products were gel-isolated and then sequenced at the London Regional Genomics Facility. Cleavage sites were determined from ABI traces, taking into account the additional A added by Taq polymerase during sequencing reactions.

### Yeast β-galactosidase reporter assay

The yeast reporter assay was performed as described (Christian *et al.* 2010). The protocol was adapted to microtiter plates. Three transformants each of YPH499 harboring the target plasmids (in pCP5.1) and YPH500 harboring the Tev-mTALENs were grown in 96-well plates at 30° overnight with shaking in synthetic complete medium lacking tryptophan and uracil (for the YPH499 target strain) or histidine (for the YPH500 Tev-mTALENs strains). The Tev-mTALEN and target strains were mated by combining 200–500 μl of target and expression strains and were incubated overnight with shaking in synthetic complete medium lacking tryptophan and uracil (for the YPH499 target strain) or histidine (for the YPH500 Tev-mTALEN strains). Cell density was measured at 595 nM by a plate reader. Cells were harvested by centrifugation, resuspended, and lysed using YeastBuster Protein Extraction Reagent (Novagen) according to the manufacturer’s protocol. A total of 60 µl of lysate was transferred to a 96-well plate and β-galactosidase activity was measured and normalized as previously described (Townsend *et al.* 2009). Miller units were normalized to a SurB dimeric FokI-TALEN or Zif268 ZFN control for assays profiling the optimal Tev-mTALEN DNA spacer length, or to the N169-T120 Tev-mTALEN on the TP15 substrate for assays profiling CNNNG cleavage site and DNA spacer requirements.

### Episomal assays in HEK 293T cells

HEK 293T cells (obtained from ATCC) were cultured in high-glucose Dulbecco’s modified Eagle medium (DMEM) supplemented with 8% fetal bovine serum (FBS) at 37° in 5% CO_2_. Approximately 2.5×10^6^ cells were seeded 24 hr prior to transfection in 6 cm plates. Cells were co-transfected with 3 μg of pExodus Tev-mTALEN and 3 μg of pcDNA3(+) TP15 target DNA using calcium phosphate and incubated at 37° with 5% CO_2_ for 16 hr before replacing media. After 48 hr, cells were harvested in phosphate-buffered saline (PBS). Plasmid DNA was isolated using the BioBasic miniprep kit. Target sites were PCR-amplified and gel-purified. After gel purification, 250 ng of each PCR product was incubated with 2 U of *Dde*I (N.E.B.) in 1× NEBuffer 2 for 1 hr at 37°. Digests were separated by electrophoresis on a 1.5% agarose gel and stained with ethidium bromide before analysis on an AlphaImager3400 (Alpha Innotech).

## Results

### Optimization of Tev-mTALEN architecture

**Figure 1:**
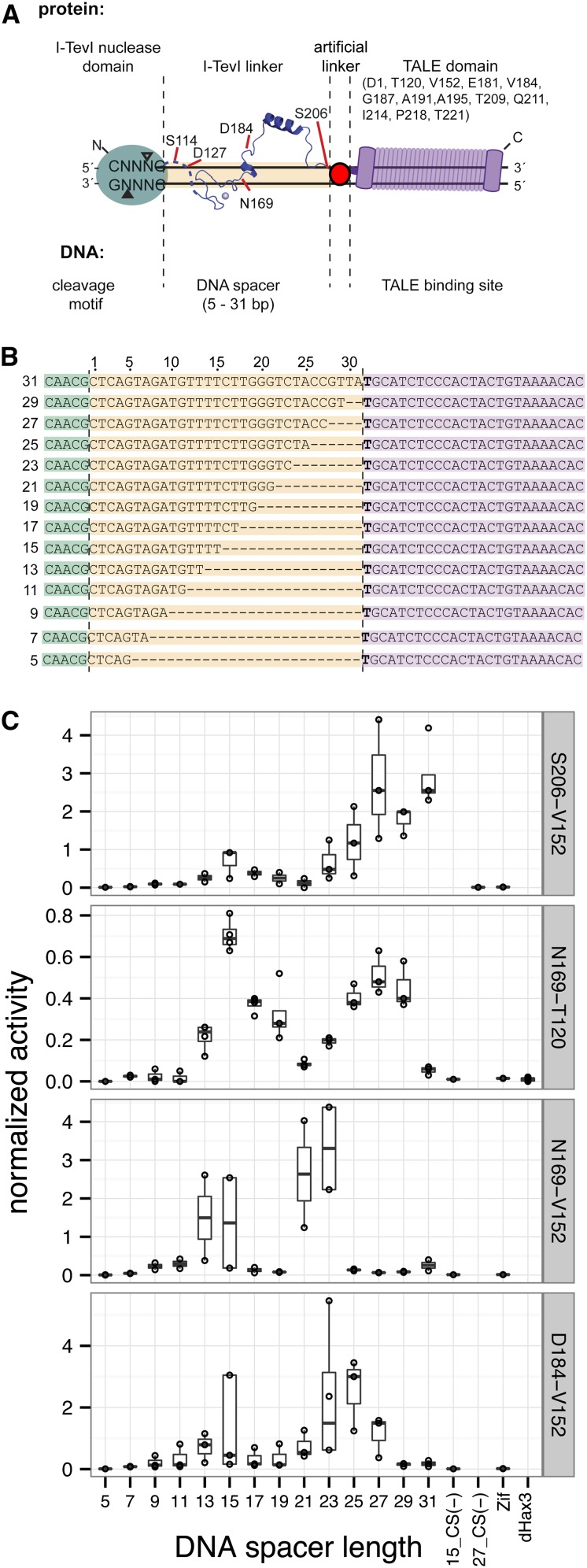
Architectures and activity of Tev-mTALENs tested. (A) Tev-mTALEN schematic highlighting the different lengths of the I-TevI nuclease domain fused to N-terminal truncation points of the PthXo1 TALE that were tested. (B) The model DNA substrates tested, derived from the phage T4 *td* gene and consisting of the wild-type 5′-CNNNG-3′ cleavage motif (CAACG), a variable length DNA spacer, and the perfect-match PthXo1 TALE binding site. (C) Boxplots of β-galactosidase activity in a yeast-based *lacZ* cleavage and repair assay. Activity is reported as a ratio of Miller units for each Tev-mTALEN to the homodimeric ZFN control, which was set at 1. The fusion points of the I-TevI fragment to the PthXo1 N-terminal residue are indicated on the right side of each plot and substrates are indicated at the bottom. The upper and lower limits of the boxes indicate the 25^th^ and 75^th^ percentiles of the data, the solid bar indicates the median of the data, and the ends of the whiskers represent 1.5-times the interquartile range. Data points are shown as black points; 15_CS(−) and 27_CS(−) are 15-bp and 27-bp DNA spacer substrates in which the preferred 5′-CAACG-3′ motif is replaced with 5′-AAACA-3′. Zif, substrate for Zif268 zinc finger nuclease. dHax3, chimeric substrate of I-TevI 15-bp DNA spacer fused to the dHax3 TALE binding site. The 15_CS(−), 27_CS(−), and dHax3 substrates were not tested with all architectures.

To determine whether the monomeric I-TevI nuclease domain remains functional when fused to TALE domains, we constructed 36 different Tev-mTALENs by fusing varying lengths of the I-TevI nuclease domain and native protein linker to the N-terminus of the TAL effector PthXo1 from the rice pathogen *Xanthomonas oryzae* pv. oryzae (Figure 1A) (Yang *et al.* 2006). Tev-PthXo1 Tev-mTALEN constructs are named using the length of I-TevI fragment, beginning at residue 1, followed by the N-terminal residue in PthXo1 (Table S1 and Figure S1). All Tev-PthXo1 Tev-mTALENs were tested against model DNA substrates derived from the phage T4 *td* gene fused to the perfect match PthXo1 binding site (Figure 1B). The TevPth (TP) substrates mimic the modularity and orientation of the Tev-mTALENs as they consist of, in the 5′ to 3′ direction, a CNNNG cleavage motif, a DNA spacer (normally contacted by the I-TevI linker), and the PthXo1 binding site. To optimize fusion architecture, we tested the activity of Tev-mTALENs on the model substrates using a quantitative yeast-based assay in which a Tev-mTALEN target site interrupts a partially duplicated *lacZ* gene (Christian *et al.* 2010). Cleavage of the target site can restore the *lacZ* gene reading frame through single-strand annealing DNA repair, resulting in β-galactosidase activity that can be normalized to benchmarked ZFNs or TALENs.

Our initial Tev-mTALEN construct consisted of residues 1-206 of I-TevI (S206) because this fragment showed high activity in the Tev-ZFE and Tev-LHE scaffolds (Kleinstiver *et al.* 2012). We found that selection of the point of fusion of this I-TevI fragment within the N-terminus of the TALE had a major effect, because fusion to the D1, T120, V152, G187, or T221 residues of PthXo1 generated nucleases with varying levels of activity (Figure 1C and Figure S2). DNA spacer length between the CNNNG cleavage motif and TALE binding site in the substrate also had a major effect. We tested spacer lengths ranging from 5 bp to 31 bp. S206-T120 and S206-V152 Tev-mTALENs displayed maximal activity on substrates with DNA spacer lengths greater than 23 bp and weaker activity on substrates with 15-bp DNA spacers.

The S206 fragment of I-TevI contains the entire region of the native I-TevI linker, including all residues that are known to make base-specific contacts to substrate in the context of the native enzyme (Van Roey *et al.* 2001). Thus, to remove potential base-specific interactions that may limit targeting (as had been done with the Tev-LHE fusion), we determined if progressively shorter lengths of the I-TevI linker could also function in the context of Tev-mTALENs. The I-TevI fragments consisting of residues 1–169 (N169) and 1–184 (D184) displayed high activity in the context of the T120 or V152 PthXo1 N-terminal fusion points, and both of these fusions also exhibited a 10-bp periodic activity on substrates with varying length spacers (Figure 1C).

Additionally, we found that deleting C-terminal residues past P1135 of the TAL domain had little effect on activity compared to analogous constructs with an intact C-terminus, except for the S206-T120 construct where the C-terminal deletion reduced activity by approximately two-fold. For the N169-T120 fusion, cleavage was directed by the TALE domain and not the I-TevI domain, because we saw no activity on a chimeric substrate consisting of the I-TevI cleavage site and 15-bp DNA spacer fused to a dHax3 TALE binding site (Figure 1C) (Mahfouz *et al.* 2012). Similarly, no activity was observed when the Tev-mTALENs were tested against the ZFN DNA substrate (Zif) Figure 1C. Collectively, the data show that fusion of different lengths of the I-TevI nuclease domain and linker to the PthXo1 TALE can create highly active Tev-mTALENs.

### Mapping of Tev-mTALEN cleavage sites

**Figure 2:**
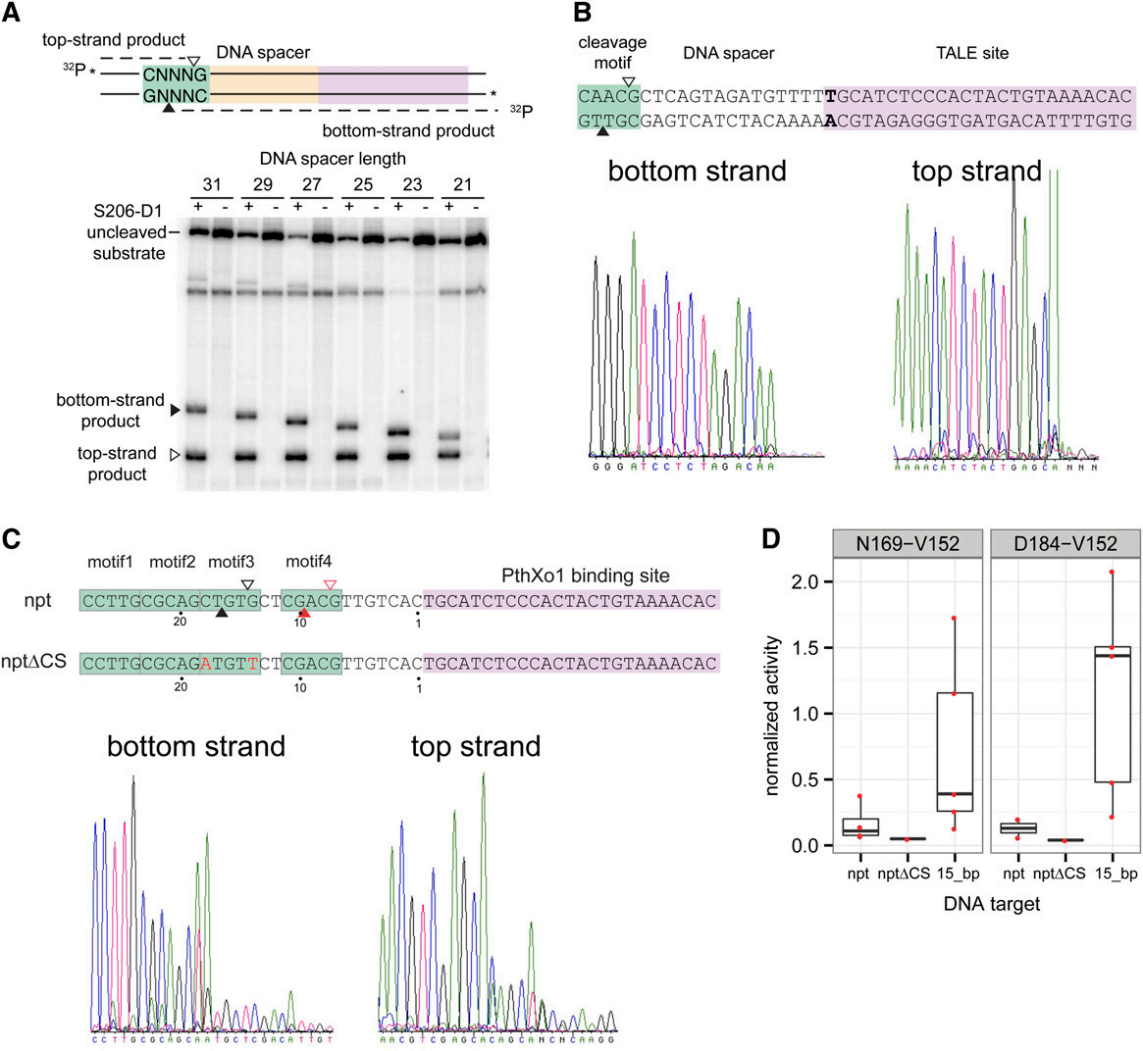
Mapping of Tev-mTALEN cleavage sites. (A) Shown at the top is a schematic of double-strand oligonucleotide substrate labeled on top-strands and bottom-strands. The top-strand nicking product is indicated by an open triangle (▵), and the bottom-strand nicking product is indicated by a filled triangle (▴). Below is a representative denaturing polyacrylamide gel of cleavage reactions with the S206-T120 Tev-mTALEN. Top-strand and bottom-strand products are represented by open (▵) and filled (▴) triangles, respectively. (B) *In vitro* mapping of N169-T120 Tev-mTALEN cleavage sites on supercoiled plasmid substrates containing a 15-bp spacer. Representative ABI traces of run-off sequencing reactions to determine the cleavage sites (taking into account that an extra "A" is added during the sequencing reactions). The cleavage sites are mapped to the complement of the sequencing trace shown. (C) *In vitro* cleavage mapping on *nptII*-PthXo1 plasmid substrates that contain four CNNNG motifs. The open (▵) and filled (▴) red triangles indicate secondary cleavage sites inferred from run-off sequencing. The electropherograms shown are derived from the *nptII* substrate. (D) *In vivo* activity of the N169-V152 and D184-V152 Tev-mTALENs on *nptII* and *nptIIΔCS* substrates (see text) measured in the yeast-based *lacZ* repair assay. Activity is normalized to a homodimeric ZFN control and is shown relative to activity on the wild-type I-TevI target with 15-bp spacer.

In general, we found that the Tev-mTALENs with the longest I-TevI fragments were most active on the substrates with longer DNA spacers. However, Tev-mTALENs with shorter I-TevI fragments, particularly the N169 and D184 constructs, displayed activity on different length DNA substrates that correlated with a 10-bp helical DNA turn. This behavior mimics that observed for the native I-TevI enzyme (Bryk *et al.* 1995; Dean *et al.* 2002) and suggests that the nuclease domain and 5′-CNNNG-3′ motif must be coordinately positioned for efficient cleavage. To map the cleavage sites of Tev-mTALENs, we used two different approaches. First, we purified the N201-D1 Tev-mTALEN and performed *in vitro* cleavage assays with oligonucleotide substrates radioactively labeled on each strand that varied in the length of the DNA spacer (from 21 to 31 bp) (Figure 2A). When resolved on a denaturing polyacrylamide gel, both the top-strand and bottom-strand products were visible. The top-strand product was a constant length because the 5′ end is always the same distance from the 5′-CNNNG-3′ cleavage motif regardless of the DNA spacer length. In contrast, the size of the bottom-strand product varied proportionally with the distance of the 5′-CNNNG-3′ cleavage motif to the TALE binding site. These results are consistent with the top-strand and bottom-strand nicks generated by Tev-mTALENs mapping to a single cleavage motif regardless of spacer length.

To extend these results, we purified the N169-T120 Tev-mTALEN and performed *in vitro* cleavage assays with a supercoiled plasmid substrate containing a target site with a 15-bp spacer. The linear product was gel-isolated and the cleavage sites were mapped by run-off sequencing. As shown in Figure 2B, the bottom (↑) and top (↓) strand nicking sites mapped to the 5′-C↑AAC↓G-3′ motif positioned 15 bp from the TALE binding site. We also constructed a substrate that consisted of the PthXo1 binding site and 28 bp of the *nptII* gene encoding neomycin phosphotransferase used in the previous cTALEN study (Beurdeley *et al.* 2013). This region of the *nptII* substrate has four CNNNG motifs, with the G of each motif located at 24 bp, 19 bp, 14 bp, and 7 bp from the TALE binding site, respectively (Figure 2C). Using purified N169-T120 Tev-mTALEN, we mapped the cleavage site to the motif at 14 bp from the TAL binding site (motif3, 5′-CTGTG-3′) (Figure 2C). This spacing of the cleavage motif from the TALE binding site agrees with our mapping data of model DNA substrates (Figure 2B). Run-off sequencing also showed evidence of weaker cleavage at the motif 7 bp from the TALE binding site (motif4). To assess the biological relevance of either motif, we used the yeast-based reporter assay to measure activity of two different Tev-mTALENs on the 28-bp *nptII* substrate and a derivative of this substrate, *nptIIΔCS*, where the C and G of motif 3 were mutated to A and T, respectively (Figure 2C). Using the N169-V152 and D184-V152 Tev-mTALENs that more accurately mimic the cTALEN architecture, we observed no more than background activity on the *nptIIΔCS* substrate and, furthermore, only low levels of activity on the wild-type *nptII* substrate compared to the native *td* 15-bp substrate (Figure 2D). These data show that the *nptII* substrate is cleaved poorly by Tev-mTALENs, that the cleavage site maps to a CNNNG motif positioned 14 bp from the TALE binding site, and that additional CNNNG motifs in the substrate cannot support robust cleavage, in contrast to the conclusions of the authors of the cTALEN study (Beurdeley *et al.* 2013).

### Defining nucleotide preference in the CNNNG cleavage motif

**Figure 3:**
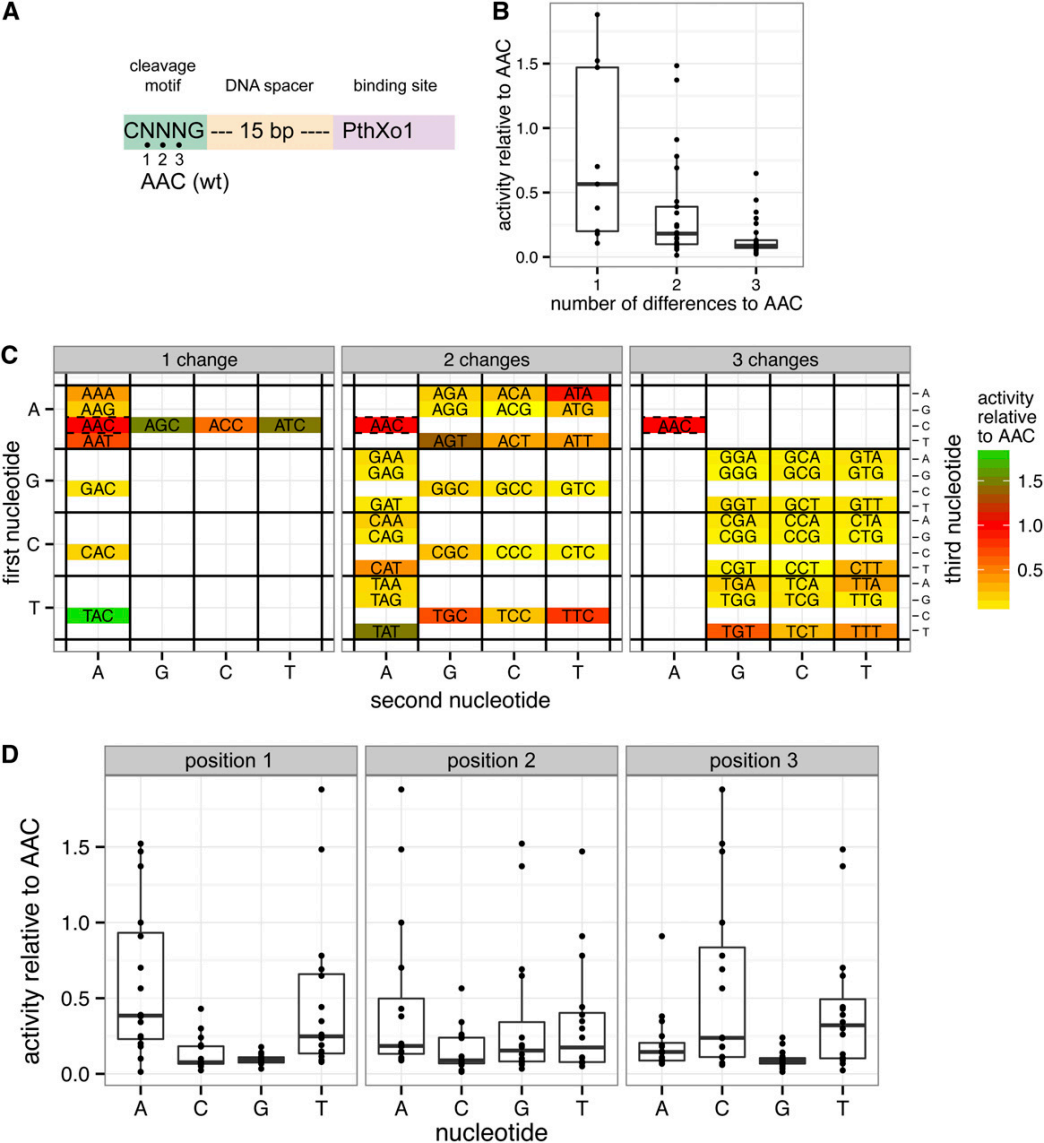
Tev-mTALEN nucleotide preferences between the C and G bases at the CNNNG cleavage site assessed using the yeast *lacZ* repair assay. (A) Schematic of the substrate used, with the randomized positions indicated and wild-type sequence shown. (B) Effect of single, double, or triple substitutions in the NNN motif on cleavage efficiency relative to the wild-type AAC sequence. Boxplots are as in Figure 1, with data points shown as dots. (C) Heatmap indicating N169-T120 Tev-mTALEN activity on individual NNN sequences, grouped according to the number of changes from the wild-type sequence, and normalized to the wild-type AAC sequence (set at 1). Axes are labeled by the first, second, and third nucleotides in the NNN sequence. The color of each motif corresponds to the median value of at least three replicates of the N169-T120 Tev-mTALEN on that substrate. For reference, the wild-type AAC motif is included as a dashed rectangle in each of the three panels. (D) Boxplots showing activity as a function of the nucelotide at the first, second, or third position across all contexts in which that nucleotide occurs at that position, relative to the wild-type motif. Black dots represent individual data points.

Accurate targeting of Tev-mTALENs requires an understanding of the tolerance of the I-TevI nuclease domain to nucleotide substitutions at the cleavage site motif. Previous studies indicated that substitutions within the central three base pairs of the cleavage motif can influence the cleavage efficiency of wild-type I-TevI (Bryk *et al.* 1993; Edgell *et al.* 2004), but whether and how they might affect cleavage in the context of a Tev-mTALEN fusion need to be addressed. Our Tev-mTALEN mapping data identified a single major CNNNG motif that supports cleavage (Figure 2), so we compared activity of the wild-type motif (CAACG) to all possible 63 variants (for AAC) in the yeast-based assay using the N169-T120 Tev-mTALEN (Figure 3A). We found that substrates with single substitutions had nearly wild-type activity, and that some single or double substitutions showed activity equivalent to or greater than the wild-type sequence (Figure 3, B and C). Yet, many variants resulted in impaired activity. Substrates with the triplets ACG, CCT, and GGG were cleaved particularly poorly (Figure 3C). When nucleotide position within the NNN triplet was considered independently of other positions, triplets with C or G at position 1 and G at position 3 showed lower activity than triplets with other bases at those positions (Figure 3D). Collectively, these data reveal tolerance to substitution at the biologically relevant CNNNG motif, consistent with previous reports of I-TevI GIY-HE cleavage specificity but, overall, A/T-rich triplets are preferred. The data also highlight the difficulty in defining a consensus cleavage site sequence, as proposed by Beurdeley *et al.* (2013), as the information regarding cleavage efficiency of individual NNN triplets is obscured in the consensus.

### Influence of the DNA spacer sequence on Tev-mTALEN activity

The I-TevI component of Tev-mTALENs consists of both the I-TevI nuclease domain (residues 1–92) and varying lengths of the I-TevI linker that presumably contact the DNA spacer region of substrate. A portion of the I-TevI linker, from residues 148 to 206, has been co-crystallized with its native DNA substrate (Van Roey *et al.* 2001). The structure reveals a linker that wraps around the minor groove of the DNA with a limited number of base-specific contacts. Through these contacts, the linker accurately positions the nuclease domain on the substrate to cleave at the 5′-CAACG-3′ motif (Dean *et al.* 2002). Previous *in vitro* cleavage assays on partially randomized substrates revealed that wild-type I-TevI can accommodate nucleotide substitutions in the DNA spacer (Bryk *et al.* 1993), yet whether the I-TevI linker can tolerate nucleotide substitutions in the context of engineered DNA-binding domains had not yet been determined.

**Figure 4:**
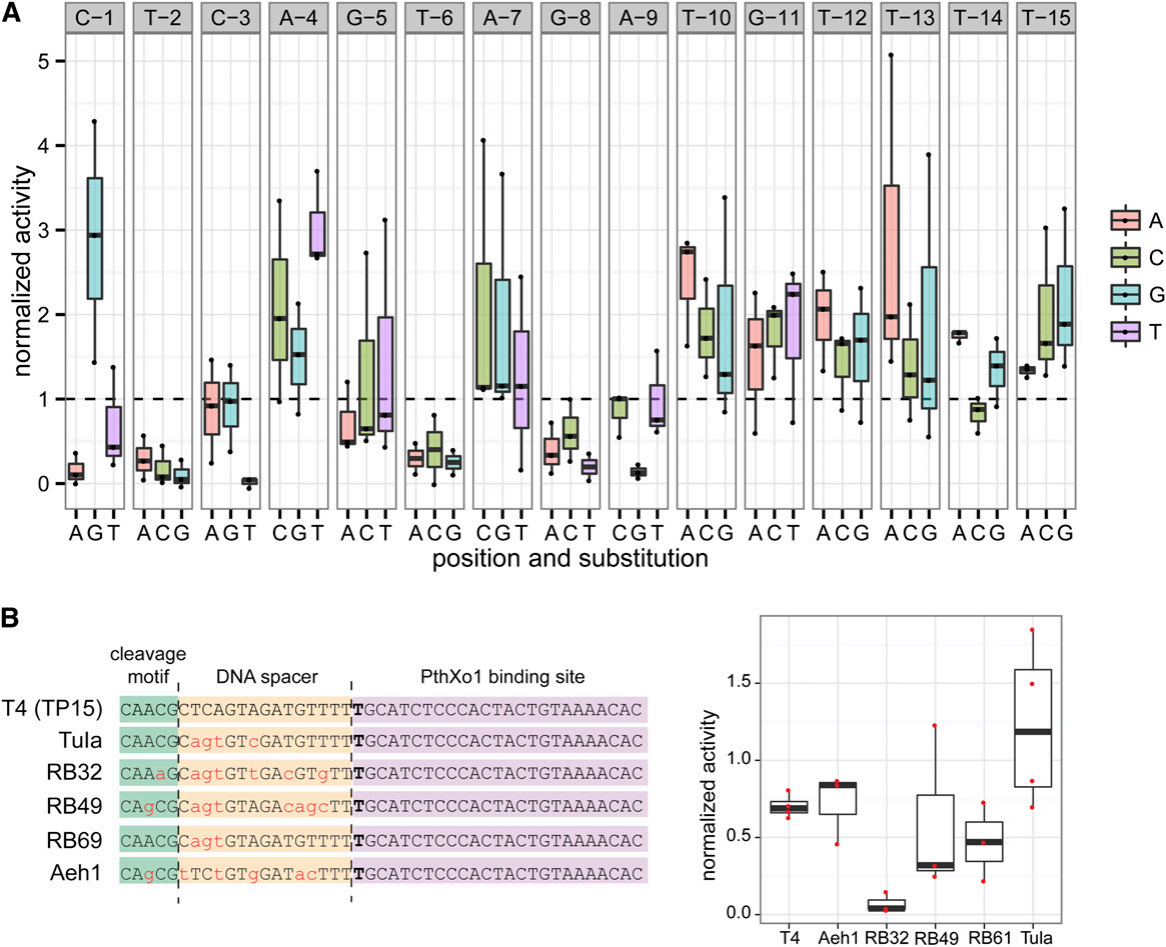
Tev-mTALEN accommodation of nucleotide variation in the DNA spacer region. (A) Boxplot of activity for 45 single nucleotide substitutions in the TP15 DNA spacer normalized to Tev-mTALEN activity on the TP15 wild-type substrate in the yeast *lacZ* repair assay. Plotted are the mean values for three biological replicates, with each biological replicate averaged from three technical replicates. The wild-type nucleotide at each position in the spacer is indicated at the top of the plot. (B) Activity on substrates derived from phage-encoded *td* genes. The substrates are shown at left, with differences in the DNA spacer and cleavage motif relative to the wild-type *td* sequence from phage T4 highlighted as lower case red letters. Activity, as in (A), is shown at right for the N169-T120 Tev-mTALEN on the different *td* substrates. Boxplots are labeled as in Figure 2.

To address this question, we used the yeast-based *lacZ* repair assay to test the *in vivo* activity of the N169-T120 Tev-mTALEN on a set of 45 substrates that encompass all possible single nucleotide substitutions at each position in the 15-bp DNA spacer (Figure 4A). The “one-off” cleavage profile revealed a substantial degree of tolerance of the Tev-mTALEN to substitution. Many positions within the DNA spacer could accept any substitution and retain activity equal to or greater than the wild-type substrate. In three positions (2, 6, and 8), however, the mean activity of each mutant substrate was below wild-type activity, although all but one of these substitutions (C3T) still resulted in activity above background.

The tolerance to single nucleotide substitutions suggested that the Tev-mTALEN could retain high activity on substrates with multiple substitutions in the DNA spacer. We tested this in two ways, both using the yeast assay. First, we generated a set of hybrid substrates in which the CNNNG cleavage motif and 15-bp DNA spacer were replaced by sequences derived from naturally occurring phage-encoded *td* genes that are 53–87% identical to the phage T4 sequence targeted by I-TevI (Figure 4B). Three of the CNNNG motifs possess single nucleotide substitutions yet are predicted to be cleavable (Figure 3C). The N169-T120 Tev-mTALEN showed similar or greater activity relative to the cognate T4 substrate on all substrates, except the RB32 *td* gene, on which it showed reduced activity.

**Figure 5:**
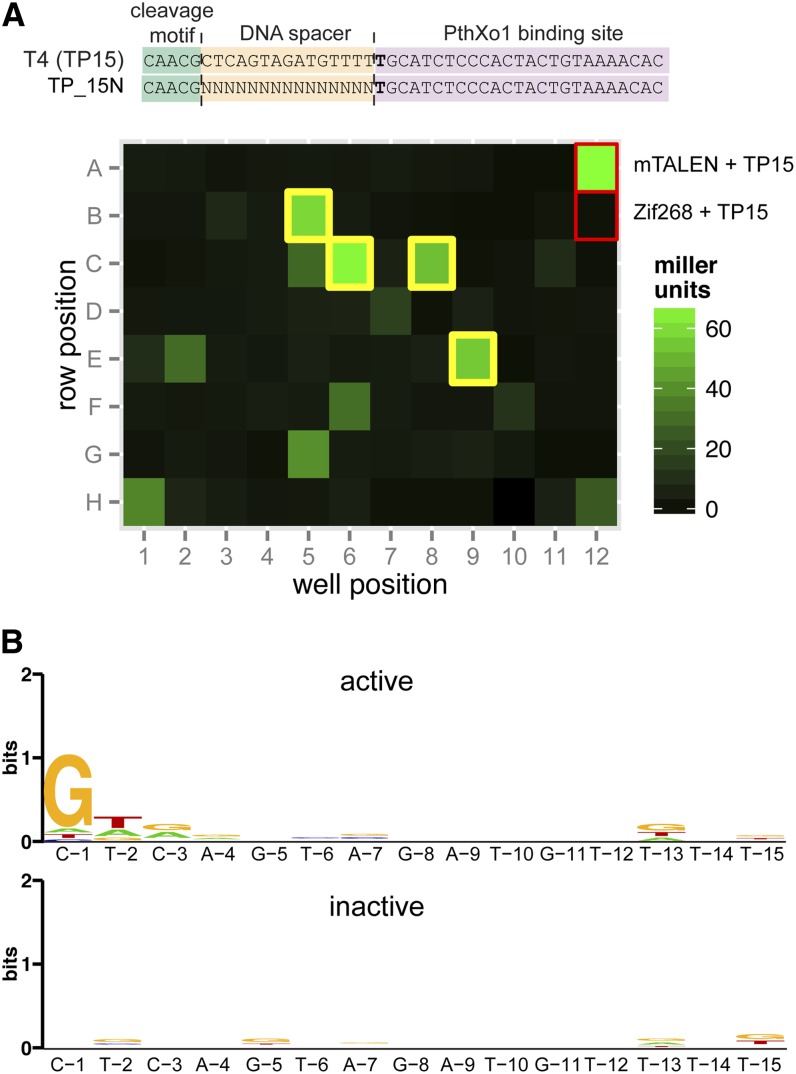
Effect of sequence variation in the DNA spacer library on Tev-mTALEN activity. (A) Activity was screened on a library of substrates with random spacer sequences, with TP_15N represented in the schematic at top relative to the wild-type T4 substrate, TP15. Shown below is a representative example of substrates tested in a 96-well microtiter plate assay in which the individual wells are colored according β-galactosidase activity (in Miller units). The red rectangles at the top right indicate the positive and negative controls. Yellow rectangles indicate substrates on which activity was greater than or within 2 SDs of the wild-type control, averaged over three technical replicates. (B) Logos plot of information content (in bits) (Schneider and Stephens 1990) per position for 49 substrates that showed high activity (cleavers) and 62 substrates with background activity (noncleavers), respectively.

Second, we generated a substrate library in which the 15-nucleotide spacer was randomized (the TP_15N library) (Figure 5A). Three hundred seventy-six independent transformants were arrayed into 96-well microtiter plates, along with a wild-type (TP15) positive control and screened in triplicate using N169-T120, along with a negative control consisting of the Zif268 ZFN against the TP15 substrate. Substrates were considered active if the mean activity of each transformant was greater than or within 2 SDs of the N169-T120/TP15 positive control (Figure S3). Inactive transformants were those with activity equivalent to background. In all, the TP_15N region was sequenced from 49 active and 62 inactive clones. The average identity for both sets of clones to the TP15 wild-type sequence was 27% (Figure S3). For the sequences derived from the active clones, strong preference was observed at position 1 (for G) and, to a lesser extent, at position 2, whereas little preference was observed at the remaining positions (Figure 5B). No nucleotide preference was observed at any position for the inactive clones. The G bias at position 1 may indicate a relaxation of the I-TevI distance constraint, whereby the linker accurately positions the nuclease domain to nick the top strand at a defined distance from the binding site. In the context of the TALE fusion, relaxation of function of the linker may allow the nuclease domain to select an alternative top-strand nicking site (defined by a critical G), and thus substrates with a G in the first position of the spacer are preferentially cleaved over substrates with other bases at this position. Although the nucleotide preferences in Figure 5B are generated from a relatively small number of sequences, they show that the I-TevI linker domain is tolerant of substitutions within the DNA spacer, in agreement with the observed cleavage of *td* sequences derived from a variety of phages (Figure 4B), but that the spacer sequence overall influences activity and position-specific nucleotide preferences occur.

### Activity of Tev-mTALENs in human cells

**Figure 6:**
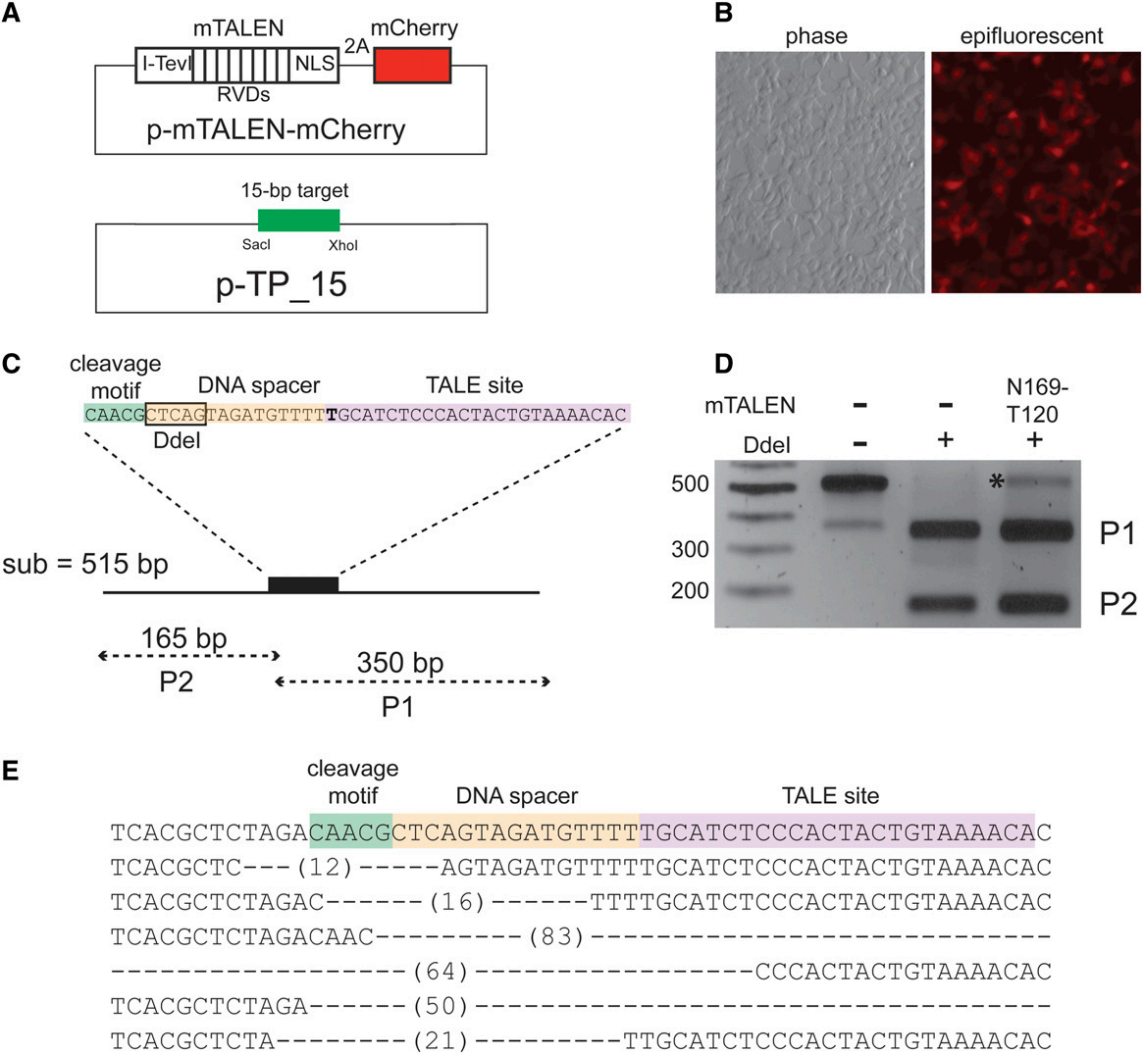
Tev-mTALEN activity in HEK293T cells on an episomal target. (A) Schematic of the vectors used for co-transfection experiments. For the expression vector, the Tev-mTALEN gene is separated from the mCherry translation reporter by a T2A peptide. (B) Example of Tev-mTALEN expression vector transfection efficiency and expression in HEK293T cells, with bright field image on the left and the epifluorescent image (1-sec exposure) of the same field of view on the right. (C) Schematic of the TP15 target, with a *Dde*I restriction site and sizes of *Dde*I digestion products indicated. (D) Agarose gel of a representative assay in which the target region was amplified by PCR from total DNA isolated 48 hr after transfection, and products were digested with *Dde*I (+) or incubated in buffer without *Dde*I (−); product resistant to cleavage by *Dde*I, which would result from mutagenic repair after cleavage by the mTALEN, is labeled with an asterisk (*). (E) Examples of mutations in the target resulting from co-transfection with N169-T120 or D184-V152 Tev-mTALENs. The *Dde*I-resistant product from (C) was cloned and several clones were sequenced. Dashes indicate the length of deletions observed in individual clones relative to the wild-type sequence.

To assess the activity of Tev-mTALENs in human cells, we cloned various Tev-mTALENs into a mammalian expression vector such that Tev-mTALEN translation can be assessed using a fused mCherry reading frame separated from the Tev-mTALEN coding sequence by a translational skipping T2A peptide (Figure 6A). Initial trials were conducted with Tev-mTALENs comprising an I-TevI sequence that was not codon-optimized for human cells expression, and we observed very weak mCherry activity indicative of poor Tev-mTALEN expression. Subsequent experiments were performed with Tev-mTALEN constructs containing human codon–optimized I-TevI, and this architecture yielded robust mCherry expression (Figure 6B).

Tev-mTALEN activity was determined in transfected HEK293T cells by co-introducing the N169-T120 construct with an episomal substrate plasmid containing the hybrid *td/*PthXo1 target with a 15-bp DNA spacer separating the CAACG motif and TALE binding site (Figure 6C). The substrate contains a *Dde*I site immediately adjacent to the I-TevI cleavage site, allowing us to estimate Tev-mTALEN cleavage efficiency as the proportion of subsequently PCR-amplified substrates that were rendered resistant to *Dde*I digestion as a result of Tev-mTALEN cleavage and nonhomologous end-joining (NHEJ)-mediated mutagenic repair. We observed ∼5–10% cleavage-resistant fragments following transfection with the substrate and Tev-mTALEN, and none was observed following transfection with substrate alone (Figure 6D). We cloned and sequenced cleavage-resistant fragments, revealing a range of deletions near the CNNNG motif (Figure 6E). Thus, Tev-mTALENs can function in HEK 293T cells to induce mutagenic DNA repair on an episomal substrate at a level of activity comparable to many reported FokI-TALENs.

## Discussion

The underlying biology of any genome-engineering reagent imposes design and targeting constraints (Halford *et al.* 2011). In the case of reagents that incorporate the FokI nuclease, the dimeric nature of the domain necessitates the design of two independent DNA-binding modules to target a single sequence (Bitinaite *et al.* 1998; Smith *et al.* 2000). Moreover, although the nonspecific nuclease activity of FokI is useful in that it does not restrict cleavage to a defined sequence, the nonspecific activity can also result in more frequent cleavage at off-target sites (Gabriel *et al.* 2011; Pattanayak *et al.* 2011). Substantial efforts have been directed to improving the FokI architecture to limit off-target cleavage, including the design of obligate heterodimeric pairs (Ramalingam *et al.* 2011; Szczepek *et al.* 2007), and the use of longer DNA-binding modules (Shimizu *et al.* 2011), such as TALEs (Christian *et al.* 2010). In the case of the CRISPR/Cas9 editing system, specificity of targeting is constrained by pairing of the RNA–DNA hybrid. As noted, use of paired Cas9 nickases addresses this problem but also has drawbacks.

An alternative approach would be to use a TALE-targeted monomeric nuclease domain such that only a single DNA-binding module would need to be designed to target a sequence for cleavage. Toward this goal, single-chain FokI TALEN variants have been developed by fusing two FokI monomers with a polypeptide linker that is then fused to the TALE domain (Sun and Zhao 2014). In contrast to FokI, use of a monomeric nuclease with some sequence specificity such as I-Tev1 might represent a further improvement, enhancing specificity by introducing moderate targeting requirements beyond those of the single TALE domain. The use of a higher-fidelity nuclease domain might be particularly appropriate for applications such as gene therapy where minimizing off-target cleavage is paramount. We have previously shown that the small, sequence-tolerant, and monomeric nuclease domain derived from the GIY-YIG homing endonuclease I-TevI could be fused to both ZFs and LHEs to create monomeric nucleases (Kleinstiver *et al.* 2012). Here, we extend the utility of the I-TevI domain by showing that it can be fused to the N-terminus of TALEs to create a programmable monomeric TALEN platform that is functional in human cells. Further, we show that the requirement of I-TevI for the motif CNNNG at the cleavage site is retained in the Tev-mTALEN context, and we provide evidence for the first time that within the CNNNG motif, A/T-rich triplets are preferred. Also, we define DNA spacer length optimal for Tev-mTALEN activity. These constraints are moderate and enhance overall specificity but must be taken into account.

Our cleavage site sequence preference results differ from those reported recently for cTALENs, which use the same I-TevI 1-184 fragment as our Tev-mTALENs (Beurdeley *et al.* 2013). A simple reason for this difference may be the CNNNG motif at which cleavage preference was tested. We mapped Tev-mTALEN cleavage and showed that the optimal position for the CNNNG motif is 15 bp upstream of the TALE binding site. In contrast, cTALEN activity was tested on variants with mutations at a motif that lies closer to the TALE binding site and that is not a site of cleavage according to our results. The cTALEN data may instead be revealing nucleotide preferences in the DNA spacer that are contacted by the I-TevI linker domain. Consistent with this notion, our (*in vivo*) spacer sequence experiments show that the I-TevI linker can tolerate substantial variation in the DNA spacer, consistent with previous *in vitro* profiling (Bryk *et al.* 1993). The data also reveal spacer sequence–dependent variation in activity with strong preference at a single position (for G at position 1) and weaker preference at another position.

Returning to the comparison with the typical FokI TALENs, another key difference of Tev-mTALENs is the orientation, because the I-TevI domain is fused to the N-terminus, in contrast to the FokI domain, which is fused to the C-terminus (Christian *et al.* 2010; Li *et al.* 2011). These fusion orientations mimic the orientation of the nuclease domain to the DNA-binding domain in the native enzymes (Derbyshire *et al.* 1997; Li *et al.* 1992). We found a wide range of activity of the Tev-mTALENs depending on the N-terminal fusion point in the TALE, possibly because certain fusion points do not correctly position the I-TevI linker and catalytic domain on the DNA. It is worth noting that some of our most active fusions are at the V152 fusion point that is commonly used in FokI-TALEN constructs (Miller *et al.* 2011), implying that the I-TevI nuclease domain could be substituted into existing TALEN assembly and expression constructs with relative ease. Interestingly, both the FokI domain and the I-TevI domain can function when fused to the opposite ends of TALEs (C-terminal for I-TevI and N-terminal for FokI), but at a much lower efficiency (Beurdeley *et al.* 2013; Li *et al.* 2011). One implication of the N-terminal I-TevI fusion to TALEs is that the same DNA sequence cannot be targeted for cleavage by the Tev-mTALENs and FokI-TALENs, making direct comparisons problematic.

In summary, our experiments demonstrate that Tev-mTALENs are a viable alternative genome-editing tool. One obvious advantage of the Tev-mTALEN platform is the monomeric nature, simplifying design requirements to target desired sequences. The moderate sequence requirements of the nuclease domain we found to be retained in Tev-mTALENs can be exploited to minimize off-targeting, or possibly to simplify constructs further by minimizing the number of TALE repeats incorporated. At the same time, however, the incomplete tolerance of Tev-mTALENs to variation in the DNA spacer between the TALE binding site and the cleavage motif that we observed highlights the need for additional study. Further progress in Tev-mTALEN-based genome editing will benefit from more extensive profiling of nucleotide interactions with the I-TevI linker domain to elucidate the basis for the influence of the spacer sequence and facilitate the development of a predictive targeting model.

## Supplementary Material

Supporting Information
