# Supplementary material for: The I-TevI Nuclease and Linker Domains Contribute to the Specificity of Monomeric TALENs
Source: G3 (Bethesda). 2014 Apr 16;4(6):1155–65. doi: 10.1534/g3.114.011445 (PMC4065259; doi:10.1534/g3.114.011445)
Supplement: Supporting Information [file supp_4_6_1155__index.html]

The I-TevI Nuclease and Linker Domains Contribute to the Specificity of Monomeric TALENs — Supporting Information 

# The I-TevI Nuclease and Linker Domains Contribute to the Specificity of Monomeric TALENs

## Supporting Information for Kleinstiver *et al.*, 2014

**Files in this Data Supplement:**

- Supporting Information - Figures S1-S4 and Table S1 (PDF, 412 KB)
- Figure S2 - mTALEN activity. (PDF, 161 KB)
- Figure S3 - Screening of randomized DNA spacer library. (PDF, 191 KB)
- Figure S4 - Example purification of an untagged Tev-mTALEN construct. (PDF, 265 KB)
- Table S1 - mTALEN constructs, named according to the length of the I-TevI fragment and the N-terminal residue of the PthXo1 TALE domain. (PDF, 114 KB)
- Figure S1 - Sequences of Tev-mTALENs. (.txt, 45 KB)
