# Supplementary material for: The I-TevI Nuclease and Linker Domains Contribute to the Specificity of Monomeric TALENs
Source: G3 (Bethesda). 2014 Apr 16;4(6):1155–65. doi: 10.1534/g3.114.011445 (PMC4065259; doi:10.1534/g3.114.011445)
Supplement: Supporting Information [file supp_g3.114.011445_011445SI.pdf]

G3·Genes | Genomes | Genetics

The I-TevI nuclease and linker domains contribute to the specificity of monomeric TALENs

Benjamin P. Kleinstiver[1#^], Li Wang[2#], Jason A. Wolfs[1], Tomasz Kolaczyk[1], Brendon McDowell[1], Xu Wang[3], Caroline Schild-Poulter[1,3], Adam J. Bogdanove[2]*, and David R. Edgell[1]*

[1] Department of Biochemistry, Schulich School of Medicine and Dentistry, Western University, London, ON, Canada N6A 5C1
[2] Department of Plant Pathology and Plant-Microbe Biology, Cornell University, 334 Plant Science, Ithaca, NY 14853
[3] Robarts Research Institute, Schulich School of Medicine and Dentistry, Western University, London, ON, Canada N6A 5B7

# these authors contributed equally to the paper
* corresponding authors

^ Present address: Department of Pathology, Molecular Pathology Unit, Massachusetts General Hospital, 149 13th Street, Charlestown, MA 02129, USA

David R. Edgell
Department of Biochemistry, Schulich School of Medicine and Dentistry, Western University, London, ON, Canada N6A 3B1
dedgell@uwo.ca
519-661-3133

Adam J. Bogdanove
Department of Plant Pathology and Plant-Microbe Biology, Cornell University, 334 Plant Science, Ithaca, NY 14853
ajb7@cornell.edu
607-255-7831

DOI: 10.1534/g3.114.011445

Figure S1 Sequences of Tev-mTALENs. Available for download as a .txt file at http://www.g3journal.org/lookup/suppl/doi:10.1534/g3.114.011445/-/DC1

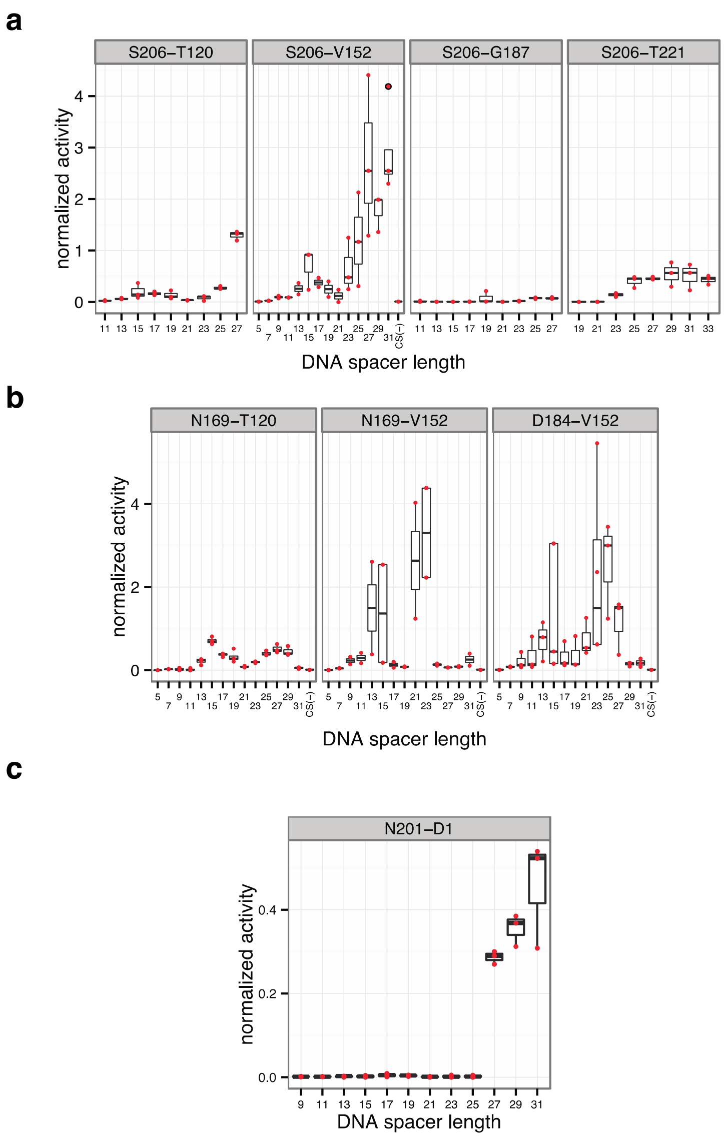

Figure S2 mTALEN activity. (A) Boxplots of β-galactosidase activity on substrates with different length DNA spacers normalized to a homodimeric ZFN control. Experiments were carried out using the constructs depicted in Figure 1. The fusion points of the I-TevI S206 fragment to the PthXo1 N-terminal residue are indicated above each set of plots. The upper and lower limits of the boxes indicate the 25th and 75th percentile of the data, the solid bar indicates the median of the data, and the ends of the whiskers represent 1.5 times the interquartile range. Data points outside of the interquartile range (outliers) are shown as black points. (B) Boxplots showing activity of shorter I-TevI fragments fused to the T120 or V152 residues of PthXo1.

a

b

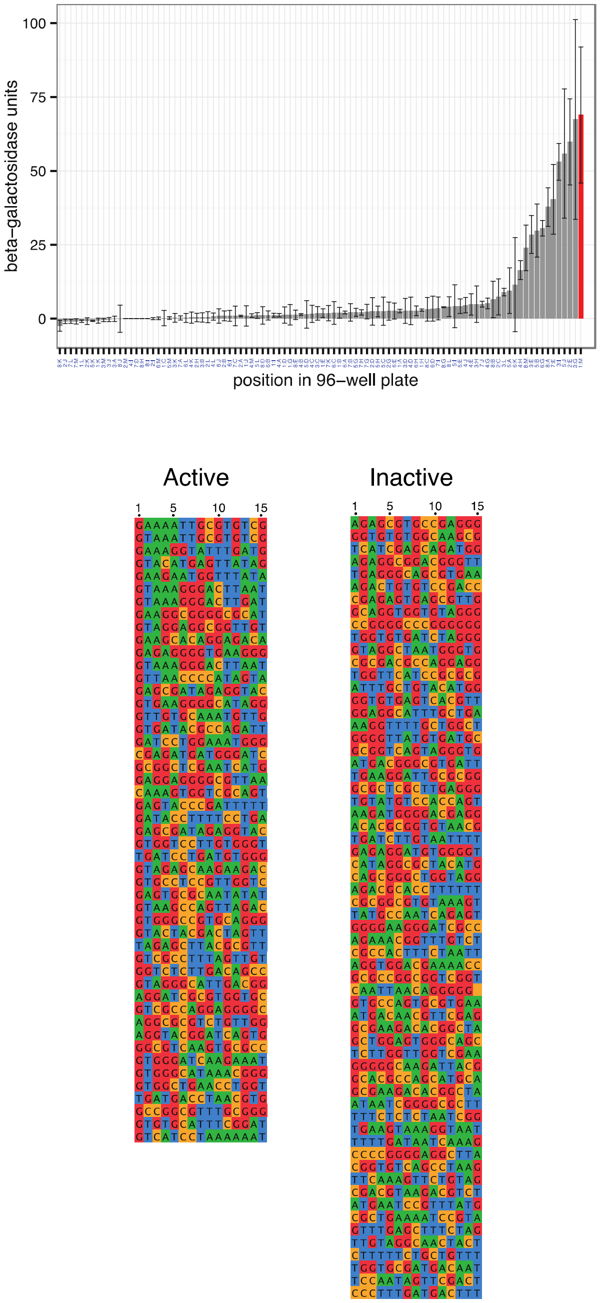

Figure S3 Screening of randomized DNA spacer library. (a) Example of ranked histogram of β-galactosidase activity for three biological replicates of one 96-well plate. The mean activity is plotted with errors bars representing standard deviation from the mean. The wild-type control (the TP15 substrate) is indicated by a red-filled bar. (b) Sequences of DNA spacers from active and inactive clones, colored by nucleotide identity.

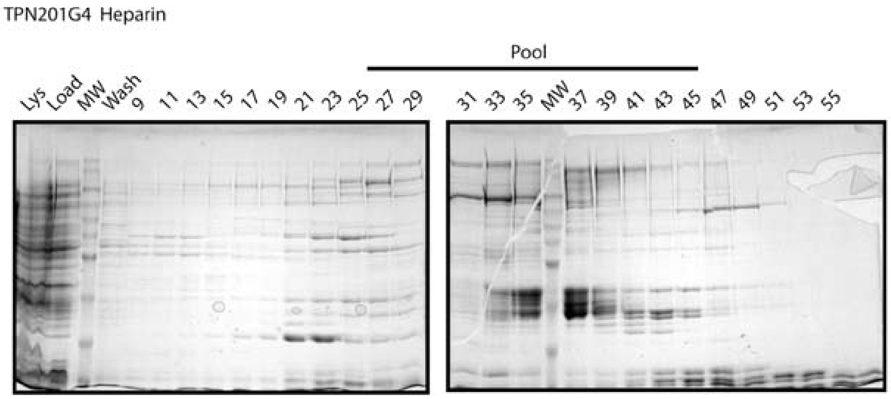

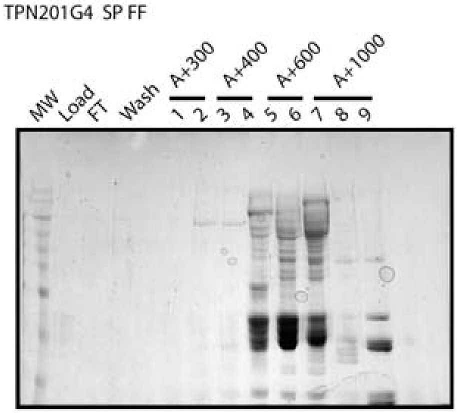

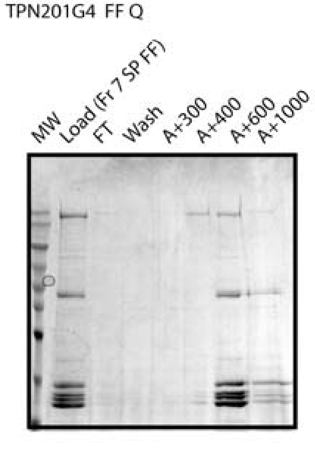

- Used the A+400 fraction
- Final Concentration for the A+400 fraction: 0.07 mg/ml
- Total Protein: 0.07 mg

Figure S4 Example purification of an untagged Tev-mTALEN construct. Shown are SDS-PAGE gels of various column fractionations.

Table S1 mTALEN constructs, named according to the length of the I-TevI fragment and the N-terminal residue of the PthXo1 TALE domain.

mTALEN	I-TevI fragment	linker	PthXo1 N-terminus	PthXo1 C-terminal truncation	active?
**S206-T221**	S206	GGGSGLQ	T221	No	Yes
**S206-T221.1**	S206	DPISRSQLQ	T221	No	Yes
**S206-T120**	S206	GGGSGLQ	T120	No	Yes
**S206-V152**	S206	GGGSG	V152	No	Yes
**S206-G187**	S206	GGGSGLQ	G187	No	Yes
**S206-G187.1**	S206	DPISRSQLQ	G187	No	Yes
**S206-T221Δ**	S206	GGGSGLQ	T221	P1135	Yes
**S206-T221.1Δ**	S206	DPISRSQLQ	T221	P1135	Yes
**S206-T120Δ**	S206	GGGSGLQ	T120	P1135	Yes
**S206-I214**	S206	None	I214	No	Weak
**S206-P218**	S206	None	P218	No	Weak
**N201-D1**	N201	GGGGGS	D1	No	Yes
**D184-V152**	D184	GGSGGS	V152	No	Yes
**N169-T120**	N169	GGSGGS	T120	No	Yes
**N169-V152**	N169	GGSGGS	V152	No	Yes
**N169-E181**	N169	GGSGGS	E181	No	No
**N169-V184**	N169	GGSGGS	V184	No	No
**N169-G187**	N169	GGSGGS	G187	No	No
**N169-A191**	N169	GGSGGS	A191	No	No
**N169-A195**	N169	GGSGGS	A195	No	No
**N169-T209**	N169	GGSGGS	T209	No	No
**N169-Q211**	N169	GGSGGS	Q211	No	No
**N169-T221**	N169	GGSGGS	T221	No	No
**N140-D1**	N140	G	D1	No	No
**D127-D1**	D127	G	D1	No	No
**D127-T221**	D127	GGGSGLQ	T221	No	No
**D127-T120**	D127	GGGSGLQ	T120	No	No
**D127-P218**	D127	None	P218	No	No
**D127-I214**	D127	None	I214	No	No
**D127-T221**	D127	GGGSG	T221	No	No
**D127-T221Δ**	D127	None	T221	P1135	No
**D127-I214Δ**	D127	None	I214	P1135	No
**D127-T221Δ**	D127	GGGSG	T221	P1135	No
**S114-D1**	S114	G	D1	No	No
