# Supplementary material for: The I-TevI Nuclease and Linker Domains Contribute to the Specificity of Monomeric TALENs
Source: G3 (Bethesda). 2014 Apr 16;4(6):1155–65. doi: 10.1534/g3.114.011445 (PMC4065259; doi:10.1534/g3.114.011445)
Supplement: Supporting Information [file supp_g3.114.011445_FigureS2.pdf]

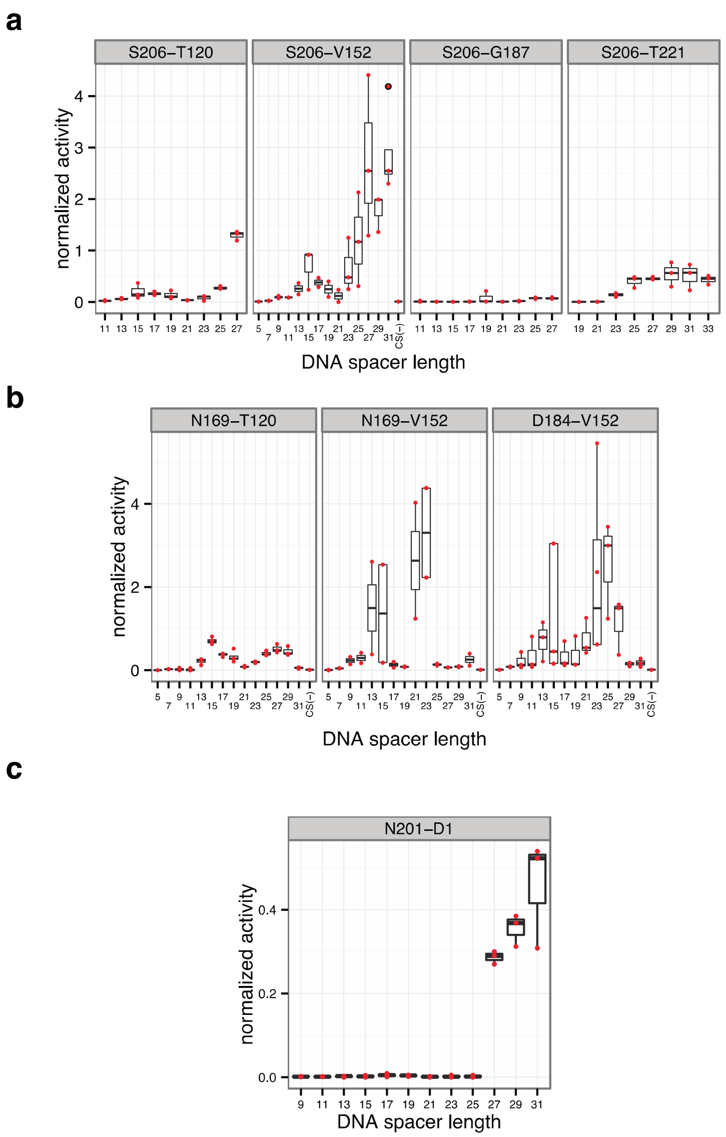

Figure S2 mTALEN activity. (A) Boxplots of β-galactosidase activity on substrates with different length DNA spacers normalized to a homodimeric ZFN control. Experiments were carried out using the constructs depicted in Figure 1. The fusion points of the I-TevI S206 fragment to the PthXo1 N-terminal residue are indicated above each set of plots. The upper and lower limits of the boxes indicate the 25th and 75th percentile of the data, the solid bar indicates the median of the data, and the ends of the whiskers represent 1.5 times the interquartile range. Data points outside of the interquartile range (outliers) are shown as black points. (B) Boxplots showing activity of shorter I-TevI fragments fused to the T120 or V152 residues of PthXo1.
