# Supplementary material for: The I-TevI Nuclease and Linker Domains Contribute to the Specificity of Monomeric TALENs
Source: G3 (Bethesda). 2014 Apr 16;4(6):1155–65. doi: 10.1534/g3.114.011445 (PMC4065259; doi:10.1534/g3.114.011445)
Supplement: Supporting Information [file supp_g3.114.011445_FigureS3.pdf]

a

b

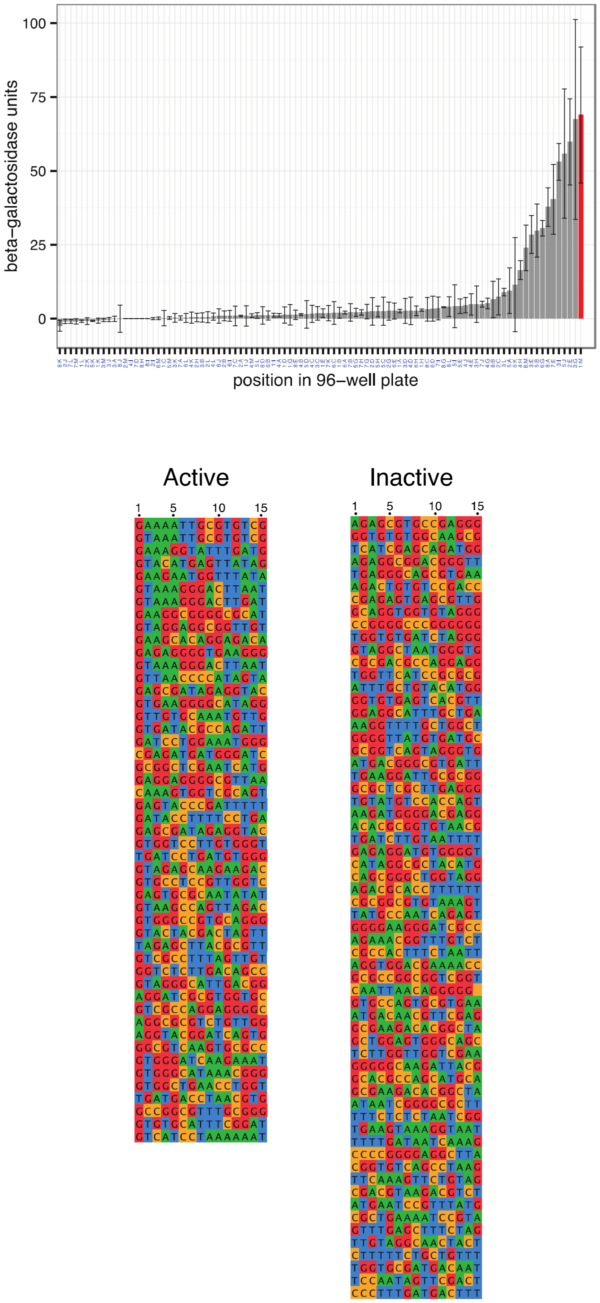

Figure S3 Screening of randomized DNA spacer library. (a) Example of ranked histogram of β-galactosidase activity for three biological replicates of one 96-well plate. The mean activity is plotted with errors bars representing standard deviation from the mean. The wild-type control (the TP15 substrate) is indicated by a red-filled bar. (b) Sequences of DNA spacers from active and inactive clones, colored by nucleotide identity.
