# Supplementary material for: The I-TevI Nuclease and Linker Domains Contribute to the Specificity of Monomeric TALENs
Source: G3 (Bethesda). 2014 Apr 16;4(6):1155–65. doi: 10.1534/g3.114.011445 (PMC4065259; doi:10.1534/g3.114.011445)
Supplement: Supporting Information [file supp_g3.114.011445_FigureS4.pdf]

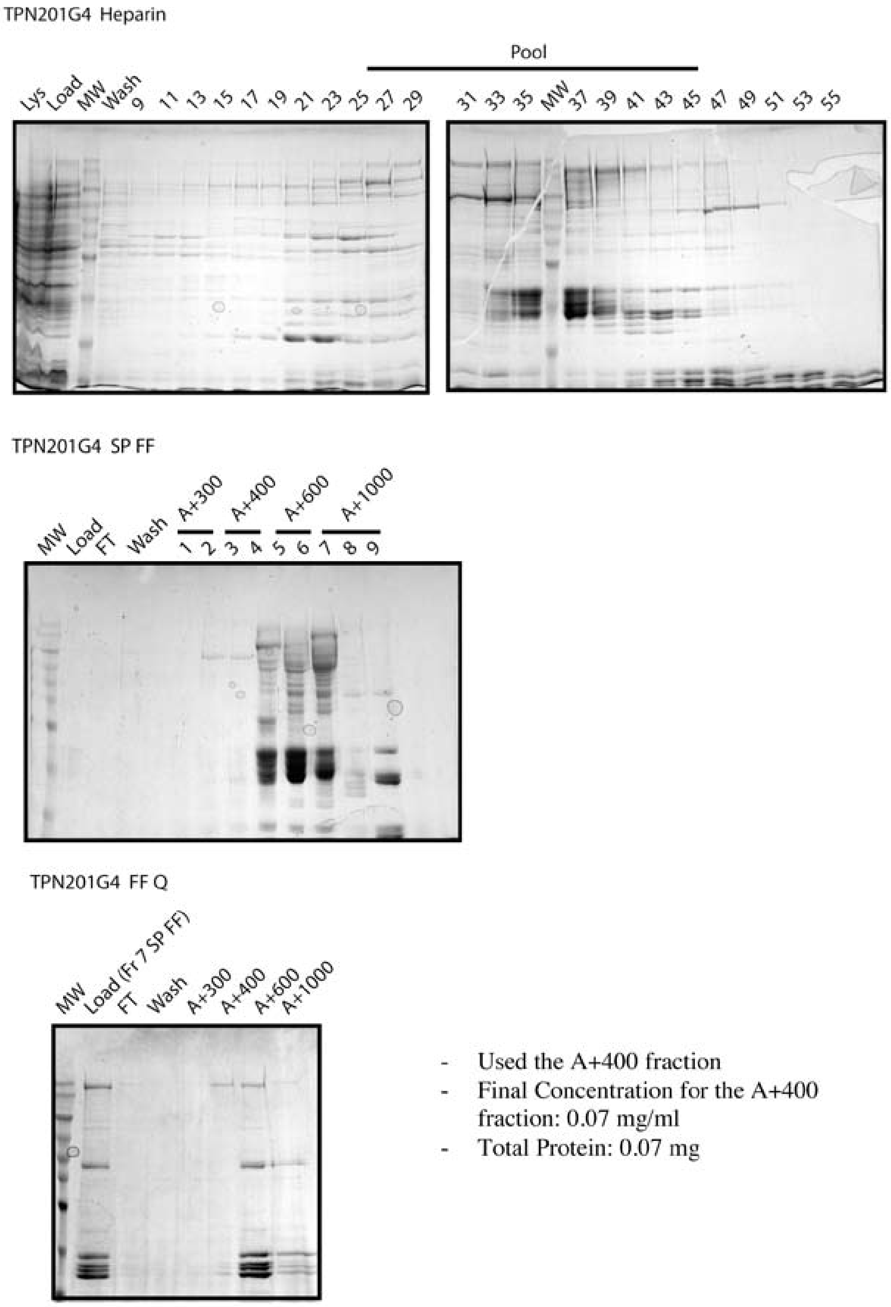

Figure S4 Example purification of an untagged Tev-mTALEN construct. Shown are SDS-PAGE gels of various column fractionations.
