# Supplementary material for: The I-TevI Nuclease and Linker Domains Contribute to the Specificity of Monomeric TALENs
Source: G3 (Bethesda). 2014 Apr 16;4(6):1155–65. doi: 10.1534/g3.114.011445 (PMC4065259; doi:10.1534/g3.114.011445)
Supplement: Supporting Information [file supp_g3.114.011445_TableS1.pdf]

Table S1 mTALEN constructs, named according to the length of the I-TevI fragment and the N-terminal residue of the PthXo1 TALE domain.

mTALEN	I-TevI fragment	linker	PthXo1 N-terminus	PthXo1 C-terminal truncation	active?
**S206-T221**	S206	GGGSGLQ	T221	No	Yes
**S206-T221.1**	S206	DPISRSQLQ	T221	No	Yes
**S206-T120**	S206	GGGSGLQ	T120	No	Yes
**S206-V152**	S206	GGGSG	V152	No	Yes
**S206-G187**	S206	GGGSGLQ	G187	No	Yes
**S206-G187.1**	S206	DPISRSQLQ	G187	No	Yes
**S206-T221Δ**	S206	GGGSGLQ	T221	P1135	Yes
**S206-T221.1Δ**	S206	DPISRSQLQ	T221	P1135	Yes
**S206-T120Δ**	S206	GGGSGLQ	T120	P1135	Yes
**S206-I214**	S206	None	I214	No	Weak
**S206-P218**	S206	None	P218	No	Weak
**N201-D1**	N201	GGGGGS	D1	No	Yes
**D184-V152**	D184	GGSGGS	V152	No	Yes
**N169-T120**	N169	GGSGGS	T120	No	Yes
**N169-V152**	N169	GGSGGS	V152	No	Yes
**N169-E181**	N169	GGSGGS	E181	No	No
**N169-V184**	N169	GGSGGS	V184	No	No
**N169-G187**	N169	GGSGGS	G187	No	No
**N169-A191**	N169	GGSGGS	A191	No	No
**N169-A195**	N169	GGSGGS	A195	No	No
**N169-T209**	N169	GGSGGS	T209	No	No
**N169-Q211**	N169	GGSGGS	Q211	No	No
**N169-T221**	N169	GGSGGS	T221	No	No
**N140-D1**	N140	G	D1	No	No
**D127-D1**	D127	G	D1	No	No
**D127-T221**	D127	GGGSGLQ	T221	No	No
**D127-T120**	D127	GGGSGLQ	T120	No	No
**D127-P218**	D127	None	P218	No	No
**D127-I214**	D127	None	I214	No	No
**D127-T221**	D127	GGGSG	T221	No	No
**D127-T221Δ**	D127	None	T221	P1135	No
**D127-I214Δ**	D127	None	I214	P1135	No
**D127-T221Δ**	D127	GGGSG	T221	P1135	No
**S114-D1**	S114	G	D1	No	No
